# Medical Opinion and Movement

**Published:** 1906-11-03

**Authors:** 


### Medical Opinion and Movement.

It will be interesting to observe how far the recent attempt to move the general practitioners throughout the country to take an interest in parliamentary and local government has succeeded. A much larger number of medical candidates than usual presented themselves this year at the municipal elections, and the returns should give some indication of the results likely to follow from these recent efforts to move the general practitioners actively to assert themselves as citizens and electors.

Christian Science seems to annihilate all moral sense on the part of some of its votaries. At the Central Criminal Court evidence was given that a Christian Science *Healer* named Riley charged one guinea a week for his attendance, that he used the letters M.D. on his card—having been a M.D. of University College, Missouri, which he had forfeited—and that he had treated the patient for lumbago, although the medical evidence showed that the deceased suffered from heart disease, and had had a large abdominal tumour for years.

A French authority, M. Legendre, has been investigating the effect of motor-car exercise on health. He is of opinion that the motor should be forbidden in cases of tuberculosis with fever, congestion of the lungs and liver, and gastric ulcer. The effect is good in certain skin affections, where rapid passage through the air produces vaso-constriction, to be followed by vaso-dilatation when the exercise is over. Motor exercise has improved cases of emphysema, nervous asthma, chlorosis, and certain alimentary affections, including gastralgia, anorexia, and chronic constipation. He urges the avoidance of motor-car journeys by patients suffering from varicose veins, epilepsy, alcoholism, and obesity. In certain forms of heart disease, too, motor exercise is to be avoided, as with such patients the results may prove serious.

The Committee of Management of the Royal Colleges of Physicians and Surgeons, London, has decided that the present medical curriculum is sufficiently long, and that they therefore decline to increase it. A considerable proportion of the students at present fail in one or more of the examinations, so that the period of study laid down as a minimum is exceeded by average students, and very much exceeded by those who are below the average. The effect of the changes in the curriculum has been gradually to decrease the number of students entering the profession of late years, and has reduced the number of candidates presenting themselves for examination; but, despite the addition of numerous degrees and diplomas in the country, the Conjoint Examining Board in England continues to attract each year to the final examination much the same proportion of students as formerly. We are glad, too, that arrangements have been made which will have the effect of providing candidates who hold the diplomas of the two Royal Colleges with a certificate in State medicine after examinations conducted by the London School of Tropical Medicine of those who have followed a course of instruction approved by the Royal Colleges.

It is seldom that our greater medical contemporaries contain such interesting reading as the current numbers for the week ending October 27th. They have published in full two important contributions—one by Professor Osler on the "Growth of Truth," to which we refer in another column, and another by Professor Clifford Allbutt on "Words and Things." There is a charm and excellence about both these contributions which should make them live in medical literature. Both professors are deservedly eminent; both have devoted active and useful lives to medical and scientific research in various fields; and both have literary gifts of no mean order. We have no doubt that the amount of time and research expended upon each of these papers was by no means small. Still the fact that they will be read by the more intelligent and active thinkers of the scientific world, as well as by thousands of other people, must in some measure compensate the authors for the personal output which their production represents. It is remarkable and encouraging to note that each address is dominated by the highest spirit. This spirit, indeed, raises the work to so high a level as to make its influence for good likely to prove of supreme importance to all conscientious workers in the scientific field now and for many years to come.

The public are rapidly coming to believe that the present London County Council lacks financial ability. Not only do its members display a great desire to vote for lavish expenditure in huge sums as a settled policy, but in smaller matters their want of financial capacity in acting as trustees for the ratepayers is evidenced more and more. The latest example of this latter financial incapacity is exhibited by the Parks Committee, who agreed on July 17th, 1906, to let to a refreshment contractor the whole of the first floor of the late Sir Spencer Wells's charming mansion at Golder's Hill, Hampstead, including the drawing-room, for the paltry sum of £15 a year. The impropriety of this wasteful policy is further demonstrated by the knowledge that the Act requires that the house in question shall be devoted to some purpose for the health, convenience, or recreation of the public. In 1900 and 1901 the house, with the unanimous approval of the London County Council, was fitted up for the use of soldiers convalescent from injuries received in the Boer War. The Parks Committee in 1903, as the result of this experiment, recommended that any houses or mansions in the Council's parks, suitable for the purpose, should be allowed to be used as convalescent homes for London children needing pure air. Why has this policy not been put into practice, and why has one of the most charming mansions near London, that at Golder's Hill, been let for £15 a year to a contractor, to the exclusion of these poor children? Londoners have a right to demand an immediate answer to these questions, and to insist upon the application of a speedy remedy for the evils in question.

